# Challenges to Identity Integration Indirectly Link Experiences of Heterosexist and Racist Discrimination to Lower Waking Salivary Cortisol in Sexually Diverse Latinx Emerging Adults

**DOI:** 10.3389/fpsyg.2020.00228

**Published:** 2020-02-25

**Authors:** Luis Armando Parra, Paul David Hastings

**Affiliations:** ^1^Department of Human Ecology, University of California, Davis, Davis, CA, United States; ^2^Center for Mind and Brain, University of California, Davis, Davis, CA, United States; ^3^Suzanne Dworak-Peck School of Social Work, University of Southern California, Los Angeles, CA, United States; ^4^Department of Psychology, University of California, Davis, Davis, CA, United States

**Keywords:** sexual diversity, Latinx, intersectionality, heterosexist discrimination, racist discrimination, identity integration, cortisol and stress

## Abstract

Heterosexist and racist discrimination may adversely impact neurobiological processes implicated in the physical and psychosocial well-being of sexually diverse Latinx people. Yet, little is known about how experiences of both heterosexist and racist discrimination are associated with adrenocortical and psychological functioning in groups of people with multiply marginalized social group identities. Through the application of the intersectionality, minority stress, and allostatic load frameworks, it was hypothesized that experiences of heterosexist and racist discrimination would be associated with disruptions to diurnal salivary cortisol patterns and challenges to identity integration. A group of sexually diverse (self-identified lesbian, gay, bisexual, and queer) Latinx emerging adults (*N* = 51; ages 18–29, *M* = 24.06 years; *SD* = 2.73) provided saliva samples and completed a series of questionnaires during a four-day testing protocol. Heterosexist and racist discrimination were both positively associated with challenges to identity integration. Challenges to identity integration, in turn, were associated with lower intercepts of diurnal cortisol slopes, and heterosexist and racist discrimination were indirectly associated with lower cortisol intercepts via challenges to identity integration. These findings suggest that experiences of heterosexist and racist discrimination may interconnect by challenging sexual and ethnic/racial identity integration and disrupting adaptive adrenocortical regulation among sexually diverse Latinx emerging adults.

## Introduction

Sexually diverse (i.e., people who self-identify as lesbian, gay, bisexual, pansexual, or queer) Latinx people experience heterosexist and racist discrimination which negatively impacts their well-being (Rhodes et al., 2020). Social environments that are not accepting or embracing of sexual and ethnic/racial diversity are pervasive stressors which may compromise multiply marginalized people’s ability to physiologically and psychosocially adjust healthfully (Parra and Hastings, 2018). The effects of pervasive social stressors are particularly concerning during emerging adulthood because this developmental period is characterized by uncertainty, heightened self-awareness (Arnett, 2000, 2001, 2004, 2006), sexual identity exploration and formation (Patterson, 2008; Morgan, 2013), and ethnic/racial identity exploration and formation (Erikson, 1968; Phinney, 2006; Kroger et al., 2010). Although most individuals navigate this developmental period successfully (Arnett, 2007), emerging adulthood also is a period of escalating mental health challenges (de Girolamo et al., 2012), particularly for vulnerable and marginalized groups of people. The role of stress physiology in the links between discrimination and identity formation during emerging adulthood for sexually diverse people of color has received little empirical attention (Parra and Hastings, 2018).

In stress frameworks applied to marginalized people, experiences of heterosexist (Meyer, 2003) and racist (Myers, 2009) discrimination constitute sources of social stress. Less adaptive adrenocortical functioning is associated with heterosexist discrimination experienced by sexually diverse people (Lick et al., 2013; Parra et al., 2016) and with racist discrimination experienced by ethnically/racially diverse people (Busse et al., 2017); both may contribute to diminished physical and mental health (McEwen, 1998; McEwen and Seeman, 1999). Heterosexist and racist discrimination also have been linked to challenges with identity integration in sexually and ethnically/racially diverse groups of people (Patterson, 2008; Umaña-Taylor et al., 2015, respectively). Sexually diverse Latinx people who have experienced both heterosexist and racist discrimination report a need to keep their sexual and ethnic/racial identities separate (Morales, 1989; Shramko et al., 2018), and this barrier to identity integration is a form of social stress associated with poor psychosocial adjustment (Sarno et al., 2015; Santos and VanDaalen, 2016; Shramko et al., 2018).

Most studies on adrenocortical functioning and identity integration in sexually and ethnically/racially diverse people have focused on single social categorical identities. These approaches rarely account for how systems and processes of racism and heterosexism are interconnected through an intersectionality framework to affect the lives of multiply marginalized people at their social locations (Crenshaw, 1989; Collins, 2015). Thus, it remains unclear how sexually diverse Latinx people internalize institutional and societal stressors and how these sources of stress may intersect and affect their physical and psychosocial adjustment. This lack of informed perspective underscores the important need for studies that examine the complexity of within-group heterogeneity of identities and experiences, using a critical application of an intersectionality lens in quantitative research (McCall, 2005; Cole, 2009; DeBlaere et al., 2010; Parent et al., 2013; Else-Quest and Hyde, 2016a, b). The overarching goal of this study was to assess associations among heterosexist and racist discrimination, adrenocortical functioning via diurnal patterns of salivary cortisol, and identity integration in a group of sexually diverse Latinx emerging adults through the integration of intersectionality, minority stress, and allostatic load frameworks.

### Heterosexism and Racism in Sexually and Ethnically/Racially Diverse Groups of People

Models of minority stress (e.g., Meyer, 1995, 2003) applied to sexually diverse groups of people suggest that heterosexism, a systemic form of oppression often expressed through acts of discrimination, constitutes a source of severe and pervasive social stress. Heterosexism is an ideology perpetuating the notion that being sexually attracted exclusively to people of the opposite biological sex assigned at birth, within a female/male binary, is superior to being sexually attracted to members of the same biological sex assigned at birth (Herek, 1996). Pervasive heterosexist discrimination has been associated with disruptions to diurnal adrenocortical activity (Parra et al., 2016). In the allostatic load (AL) framework (McEwen, 1998; McEwen and Seeman, 1999), pervasive social stressors can repeatedly evoke and tax neurobiological stress responses and thereby confer risk for poor health. Moreover, heterosexist discrimination also is associated with poor psychosocial adjustment, such as challenges to sexual identity integration (Patterson, 2008; Morgan, 2013). Sexual identity integration is a dynamic process of empowerment through the adoption of positive attitudes and beliefs about one’s marginalized sexual identities and the disclosure of one’s sexual identities to others (i.e., outness; Rosario et al., 2001). Challenges to sexual identity integration are associated with negative self-perceptions such as internalized homonegativity (faulting oneself for being non-heterosexual) (Igartua et al., 2003; Herek and Garnets, 2007; Herek et al., 2009).

Models of minority stress applied to ethnically/racially diverse groups of people (Myers, 2009) also posit that racism is a systemic form of oppression that can be enacted through discrimination and constitutes a severe and pervasive form of social stress (Paradies et al., 2015). Racism refers to beliefs and actions that evaluate a person’s non-majority cultural heritage or a person’s non-majority external social classification based on phenotype, language, or membership in an ethnic/racial group as inferior to, or less than, that of a majority group member (Jones, 2001). Pervasive racist discrimination has been associated with challenges to ethnic/racial identity integration (Pahl and Way, 2006; Umaña-Taylor et al., 2015) and with alterations to typical daily patterns of salivary cortisol (Busse et al., 2017). Ethnic/racial identity integration refers to an ethnically/racially diverse person’s sense of belonging to their cultural heritage (Phinney and Ong, 2007; Quintana, 2007) and ethnic/racial group (Umaña-Taylor, 2004). Both challenges to ethnic/racial identity integration (Umaña-Taylor et al., 2015) and dysregulated daily patterns of salivary cortisol production (Busse et al., 2017) in the context of racism are associated with health disparities in groups of Latinx people.

Models of minority stress applied to the study of sexual and ethnic/racial group membership often examine people who identify with these social groups as if their memberships are based on discrete and separable marginalized social categorical identities (Parra and Hastings, 2018). For example, a queer Latinx person typically would be examined as queer *or* Latinx, in the context of heterosexism *or* racism, respectively, but not at their different locations of an intersection (e.g., queer Latinx in the context of interacting systems of oppression). Systems and institutions, including science, often use these single categorical identity-focused approaches, which render invisible the comprehensive lived experiences of people who belong to multiply marginalized social groups affected by multiple systems and forms of oppression (Crenshaw, 1989).

Scientists have called for a broader intersectional perspective to accommodate quantitative methods across scientific disciplines (McCall, 2005; Bowleg, 2008; Cole, 2009; Else-Quest and Hyde, 2016a, b) and have emphasized a critical distinction between additive and multiplicative *approaches* and *effects* (see Else-Quest and Hyde, 2016a, b for full review). Additive and multiplicative *approaches* assume that multiply marginalized people’s experiences of various forms of oppression can be captured by treating each marginalized social categorical identity and/or experience independently (single-axis; e.g., being queer *or* Latinx), additively (e.g., being queer *and* Latinx), or multiplicatively (e.g., being queer *x* Latinx). These approaches have been called “antithetical to intersectionality” (Else-Quest and Hyde, 2016a, p.162; also see McCall, 2005; Collins, 2015) because they assume that each social categorical identity is discrete and independent of social structures that perpetuate power and privilege. Yet, additive and multiplicative *effects* (as statistical main and interaction effects, respectively) are inherent to quantitative analyses (Bowleg, 2008; Else-Quest and Hyde, 2016a, b). Additive and multiplicative effects are presumed to measure the unique and complex contributions of specific and intertwined marginalized social categories to help explain social inequalities and inequities, within nested systems of power and privilege, at varying social locations (e.g., health disparities specific to queer Latinx people) (McCall, 2005; Bowleg, 2008; Else-Quest and Hyde, 2016a, b). Additive and multiplicative statistical effects are considered viable when integrating an intersectionality lens with quantitative methods because they may effectively measure how two or more systems of oppression intersect and structurally, institutionally, and interpersonally disempower and confer risk to multiply marginalized people (Bowleg, 2008; Cole, 2009).

### Discrimination and Identity in Sexually Diverse Latinx Emerging Adults

Identity integration is an essential task during emerging adulthood that is critical for psychosocial adjustment (Erikson, 1968; Marcia, 1987; Arnett, 2000, 2001, 2004, 2006). Sexual identity integration (Rosario et al., 2001) and ethnic/racial identity integration (Phinney, 2003, 2006; Sellers et al., 2003) promote well-being and protect against discrimination. Navigating multiply marginalized social group categorical identities is considered an exploratory and challenging process of identity integration for many people and may be particularly complex and stressful for sexually diverse people of color (Morales, 1989; Espín, 1993). Sexual and ethnic/racial identity integration for sexually diverse people of color may encompass consolidating opposing cultural beliefs and expectations as well as managing the effects from experiences of heterosexist discrimination within their majority-heterosexual ethnically/racially diverse communities and racist discrimination within the mainstream majority-White sexually diverse community (Morales, 1989; Espín, 1993; Meyer, 2010; Balsam et al., 2011; Morales, 2013). Difficulties with or challenges to multiple identity integration may be a mechanism by which heterosexist and racist discrimination marginalize sexually diverse Latinx people to affect their adrenocortical stress responses. Processes specific to multiple identity exploration and formation are particularly salient during emerging adulthood (Arnett, 2004) and integrating multiple marginalized social group memberships in heteronormative and racist social contexts can be an ongoing challenging process (Morales, 1989; Espín, 1993) which has been implicated with health inequities (e.g., Shramko et al., 2018). Thus, emerging adulthood may be marked by particular stressors for sexually diverse Latinx individuals that disrupts integration of their multiple marginalized identities.

Although the minority stress model predominantly focuses on single categorical social identities specific to sexual diversity (e.g., lesbian, gay, bisexual; Meyer, 2003), it also acknowledges that sexually diverse people of color may further experience unique stressors associated with their ethnicity/race (i.e., sexually diverse people of color experience both heterosexism and racism; Meyer, 2010). Sexually diverse Latinx people experience heterosexist discrimination based on their sexual orientation within Latinx communities (Espín, 1993; Díaz et al., 2001, 2006; Ryan et al., 2009) and racist discrimination based on their ethnicity/race within majority-White sexually diverse communities (Morales, 1989; Balsam et al., 2011). Sexually diverse Latinx emerging adults who face discrimination based on their marginalized sexual and ethnic/racial identities report feeling less supported by others, have lower self-esteem, have more depression and anxiety symptoms, and are at elevated risk for suicide (see Rhodes et al., 2020 for full review). The intersectionality framework provides a comprehensive lens by which to examine the lived experiences of multiply marginalized people (Crenshaw, 1989). Thus, the integration of an intersectionality framework and the minority stress model can bring visibility to and a richer contextualization of how heterosexism intersects with racism to disadvantage sexually diverse Latinx people (Parra and Hastings, 2018).

### Adrenocortical Markers of Stress Regulation in Response to Heterosexist and Racist Discrimination

Adrenocortical functioning may be adversely affected by heterosexist and racist discrimination that is targeted toward sexually diverse Latinx people. The allostatic load (AL) framework suggests that chronic or extreme stressors undermine the neurobiological systems that support individual and social adaptation through flexible allocation of physiological resources (allostasis; McEwen, 1998; Goldstein and McEwen, 2002), leading to AL (McEwen and Stellar, 1993). The hypothalamic-pituitary-adrenal (HPA) axis is one of the body’s primary stress response and regulation systems (Gunnar and Adam, 2012) and often is used as a marker of AL (Hastings et al., 2011). The HPA axis is active throughout the circadian cycle; the normative diurnal pattern of cortisol production is established early in life and is characterized by high cortisol concentrations at waking, followed by a brief increase in HPA activity for approximately 30–45 min, then decreasing cortisol levels throughout the day to their lowest levels in the evening (Gunnar and Adam, 2012). This diurnal pattern is typically quantified across multiple saliva samples collected over one or more days to derive a salivary cortisol intercept (baseline waking cortisol) and slope (changes in cortisol from waking to evening) (Hruschka et al., 2005).

High waking cortisol levels, or higher cortisol intercepts, have been associated with more life satisfaction and self-acceptance, and with less anxious arousal, anticipatory stress, and depression (Vargas and Lopez-Duran, 2014; Rector et al., 2019). Exposure to pervasive stress has been associated with lower cortisol intercepts (Adam and Kumari, 2009). Steeper or more negative diurnal cortisol slopes are considered normative, allowing the body to readily allocate resources for self-regulation (Gunnar and Adam, 2012). Flatter or less negative diurnal slopes, which are associated with chronic or pervasive exposure to stressors, also have been implicated with poorer health and psychosocial adjustment (Juster et al., 2010; Guerry and Hastings, 2011; Lick et al., 2013). Whether a flatter slope reflects hypercortisolism (elevated HPA activity producing maladaptively high circulating cortisol) or hypocortisolism (diminished HPA activity producing maladaptively low circulating cortisol) depends in part on whether the cortisol intercept is higher or lower, respectively.

Pervasive and severe social stressors have been associated with both HPA axis hyperactivity (Busse et al., 2017) and HPA axis hypoactivity (Adam, 2012). Experiences of heterosexist discrimination have been associated with higher and flatter diurnal cortisol slopes, with flatter diurnal slopes mediating the link between heterosexist discrimination and depressive symptoms in a group of LGB emerging adults (Parra et al., 2016). Conversely, the association between discrimination and lower cortisol intercepts (waking cortisol) has been documented in studies of ethnically/racially diverse groups of people. In a study of ethnically and racially diverse late adolescents, which included Latinx people, experiences of racist discrimination were associated with lower waking cortisol and flatter salivary cortisol slopes (Huynh et al., 2016). Discrimination and flattened diurnal cortisol slopes also were associated in a sample that included Latinx emerging adults (Zeiders et al., 2014). These studies of discrimination and diurnal cortisol patterns suggest that experiences of ethnic/racial and heterosexist discrimination during emerging adulthood may be associated with either atypically elevated or diminished adrenocortical activity, as evidenced by either high or low cortisol intercepts paired with flattened diurnal slopes.

In our review of the literature, there is only one published study on diurnal adrenocortical functioning at the intersection of sexual orientation and racial identities. Cook et al. (2017) reported that Black bisexual and gay men had flatter diurnal cortisol output, reflecting less decline (i.e., shallower diurnal slope) and higher evening cortisol levels when compared to White bisexual and gay men. Our current study builds on Cook et al. (2017) by assessing actual measurement of heterosexist and racist discrimination instead of only using marginalized social categorical identities. Although it has been important and informative for researchers to apply psychosocial and neurobiological methods to studies with multiply marginalized populations, intersectionality scholars have critiqued the use of social categorical identifiers as proxies for experiences of oppression without assessing actual experiences (e.g., Bowleg, 2008; Cole, 2009; Parent et al., 2013). A comparative or between-group approach that does not consider within-group heterogeneity presumes similar or identical life experiences for all individuals who share those social group memberships and social locations, which may limit our understanding of how complex and multi-faceted contexts of discrimination are linked with physiology (Parra and Hastings, 2018).

### The Impacts of Heterosexist and Racist Discrimination on Sexual and Ethnic/Racial Identity Integration in Sexually Diverse Latinx Emerging Adults

The integration of sexual orientation and ethnic/racial identities is a process unique to sexually diverse people of color, involving the experience and resolution of tensions between their marginalized sexual and ethnic/racial identities, which Morales (1989) referred to as conflicts in allegiances (CIA). Morales argued that sexually diverse people of color’s awareness of their social belonging to both a marginalized sexually diverse group and a marginalized ethnically/racially diverse group was associated with a need for these identities to remain separate. Both heterosexist and racist discrimination have been shown to engender challenges to identity integration as measured through CIA (Morales, 1989; Sarno et al., 2015; Santos and VanDaalen, 2016). Sexually diverse people of color with strong ethnic/racial group orientation report more group identity conflict and less engagement in sexually diverse communities (Sarno et al., 2015) and high group identity conflict has been associated with poor psychosocial adjustment (Santos and VanDaalen, 2016). Shramko et al. (2018) showed that sexually diverse Latinx emerging adults who reported more heterosexist and racist discrimination struggled with integrating their sexual and ethnic/racial identities when compared to sexually diverse Latinx emerging adults who reported less heterosexist and racist discrimination. Thus, assessing challenges to integration of sexual and ethnic/racial identities using an inherently intersectional approach may reveal how heterosexist and racist discrimination concurrently shape the adrenocortical and psychosocial adjustment of sexually diverse Latinx emerging adults.

### Hypotheses

In the current study, we tested the associations between single-axis measures assessing heterosexist and racist discrimination, an inherently intersectional measure of challenges to identity integration, and diurnal salivary cortisol intercepts and slopes in a sample of sexually diverse Latinx emerging adults. We hypothesized that more experiences of heterosexist and racist discrimination (additive effects), including the statistical interaction between the two (i.e., heterosexist X racist discrimination; multiplicative effects) would be associated with (1) lower salivary cortisol intercepts and/or flatter slopes and (2) more challenges to identity integration. These first two hypotheses were important to establish whether single-axis measures of discrimination, as additive and multiplicative effects, were associated with identity integration and adrenocortical functioning of sexually diverse Latinx people. Further, we hypothesized that more challenges to identity integration (3) would be associated with lower cortisol intercepts and/or with flatter cortisol slopes and (4) would function as a mechanism accounting for the associations of heterosexist and racist discrimination with cortisol intercepts and slopes.

## Materials and Methods

### Participants

Two hundred and two emerging adults (*N* = 202) (*n* = 101 sex-assigned female at birth) between the ages of 18–29 (*M* = 23.13 years, *SD* = 2.59) and self-identifying as sexually diverse Latinx (*n* = 51), sexually diverse White (*n* = 51), straight Latinx (*n* = 49) or straight White (*n* = 51) were recruited in Northern California through free and paid advertisements on social media, including flyers distributed at information booths during Pride events. To test the specific hypotheses of the current study, only the sexually diverse Latinx group (*N* = 51; *n* = 27 sex-assigned female at birth; *M* = 24.06 years; *SD* = 2.73) were included. Sexually diverse Latinx participants self-identified as bisexual/pansexual (33.3%) (*n* = 17), gay (35.3%) (*n* = 18), lesbian (5.9%) (*n* = 3), and queer (25.5%) (*n* = 13); and as monoethnic/racial Latinx (82.4%) (*n* = 44) and biracial Latinx-White^1^ (17.6%) (*n* = 9). Participants reported their gender as cisgender female (43.1%) (*n* = 22), cisgender male (45.1%) (*n* = 23), transgender female-to-male (5.9%) (*n* = 3), and as genderqueer or non-binary (5.9%) (*n* = 3). The sample consisted of employed (57%) and unemployed (6%) community members, college students (27%), and people who reported being both employed and a college student (10%). Education levels included having a college degree attained (45%) or in progress (45%), vocational training certificate attained (4%), and high school completed (6%). Most of the sample reported an annual income less than $20k (57%), with the balance reporting $20 to $30k (8%), $30 to $40k (22%), and more than $40k (13%).

### Procedure

Participants completed a 4-day testing protocol after being phone screened for eligibility based on the following criteria: self-identification as lesbian, gay, bisexual, queer, or straight; monoethnic/racial Latinx, monoethnic/racial White, or biracial Latinx-White; fluent in English; and between the ages of 18–29 years. Participants who reported taking antidepressants known to normalize salivary cortisol levels were invited to participate because one of the overarching goals of the larger study focused on mental health, and including participants using antidepressants would make these analyses more generalizable (see Deuschle et al., 2003, for a review). Participants who reported the use of hormones (e.g., birth control, estradiol, and testosterone), glucocorticoids^2^, and other medications also were enrolled in the study. The use of these medications was tested for their associations with raw salivary cortisol concentrations prior to log transformations and computing intercepts and slopes for the main analyses (see section “Results”).

#### Visit 1

After the phone screen, participants met with research staff. During the first visit, participants gave informed consent and completed a questionnaire. Participants practiced saliva sampling and were instructed to collect three saliva samples per day over two consecutive weekdays (for a total of 6 samples) using absorbent oral swabs (Salivettes^TM^, Salimetrics Inc., State College, PA, United States). Participants reported their usual times of waking and going to bed on weekdays, which also were their expected times of waking and going to bed on the two specific weekdays they selected for in-home data collection. Research staff programmed automated text messages to participants’ phones and automated email reminders synchronized to their reported wake time, 30 min post waking, and at bedtime. This approach was used to capture each participant’s own circadian rhythm indexed by their self-reported waking and bedtimes (the 30-min post waking samples were not included in the current analyses of diurnal cortisol intercept and slope). Participants received printed instructions, a freezer ice pack, and a second questionnaire packet that included daily diaries for the saliva collection protocol. Participants received a $25 cash payment at the end of the visit.

#### Self-Administered Procedures at Home

Participants were instructed to not brush their teeth for 2 h before, and to not consume food, drinks, tobacco, or caffeine for 1 h before, collecting each saliva sample. To help participants adhere to the sampling protocol, participants received text messages and email reminders. Participants recorded the specific time and date when each sample was taken. Saliva samples were kept in freezer ice packs at the participants’ home freezers. Participants used daily diaries to log whether they had experienced unusual events, or had consumed food, drinks, tobacco, or caffeine 1 h prior to completing each sample. The exact sampling collection times varied across participants in order to conform with each participant’s circadian cycle (Adam and Gunnar, 2001). Participants completed a second questionnaire packet.

#### Visit 2

Participants returned their frozen saliva samples in the icepacks and their second questionnaire to research staff. At this time, research staff checked the questionnaire packet and daily diaries for completion. Participants were thanked for their work, debriefed, and received a $75 cash payment. In sum, participants were compensated $100 in cash for their time and efforts.

#### Diurnal Cortisol Assays

Diurnal cortisol was assayed from the saliva samples. Saliva samples were stored in a medical freezer at −30°C for 2 months in the laboratory until all data were collected to assay cortisol in one batch. Samples were shipped to Salimetrics Saliva Lab (Salimetrics, Carlsbad, CA, United States) for salivary cortisol assay, in duplicate, using a highly sensitive enzyme immunoassay (Salimetrics, State College, PA, United States). The minimum detection ranged from 0.007 to 1.8 μg/dL, and the intra- and interassay variabilities were 8.31 and 7.69%, respectively. The averages of the duplicate cortisol values were used in all subsequent analyses.

### Measures

#### Demographic Information

Participants reported demographic information including their age, biological sex assigned at birth, gender, sexual orientation, ethnicity/race, education, employment status, and household income.

#### Heterosexist Discrimination

The Revised Gay-related Stressful Life Events scale (Rosario et al., 2002) is a 12-item measure used to assess heterosexist discrimination, for example, *“being physically assaulted in a gay-bashing incident”; “getting in trouble with the police because of your sexual orientation.”* Response options were binary, *Yes* = *1*, *No* = *0.* Two items were removed due to zero variability across responses. Hence, the possible range for this scale was 0–10, with higher summed scores suggesting more stressful events related to heterosexist discrimination. Tetrachoric inter-item correlations were computed to derive an ordinal alpha (Gadermann et al., 2012) an index of internal consistency of the binary response options, Ordinal alpha α = 0.80.

#### Racist Discrimination

The Brief Perceived Ethnic Discrimination Questionnaire – Community Version (Brondolo et al., 2005) is a 17-item scale used to assess racist discrimination^3^. All items in the scale were used to compute racist discrimination (Brondolo et al., 2005), for example, “*Have others actually hurt you and tried to hurt you (e.g., kicked or hit you)?*”; *“Have policemen or security officers been unfair to you?”* Response options for each item ranged from *1* = *Never* to *5* = *Very often*. Higher averaged scores suggested more experiences with racist discrimination, Cronbach’s alpha α = 0.93.

#### Challenges to Identity Integration

The Conflicts in Allegiances (CIA; Sarno et al., 2015) questionnaire is an inherently intersectional measure of a unique form of social stress experienced by sexually diverse people of color. The six-item CIA scale captures the degree to which people who hold memberships and allegiances to multiple marginalized social groups experience challenges to integrating their sexual and ethnic/racial identities, for example, *“I have not yet found a way to integrate being LGBQ with being a member of my cultural group”*; *“I feel as if my sense of cultural identity is at odds with my LGBQ identity.”* Response options for each item ranged from *1* = *Strongly disagree* to *7* = *Strongly agree*. Two items were reverse coded. Higher averaged scores suggested more challenges to identity integration, Cronbach’s alpha α = 0.82.

### Cortisol Intercepts and Slopes

Raw biomarker hormone values were log_10_ transformed to correct for positive skewness.

Use of medications, reported experiences of unusual events, and consumption of food, caffeine, or tobacco 60 min before each sample were dummy coded (*No* = *0, Yes* = *1*) and tested for their respective associations with raw cortisol values^4^. Using multilevel linear modeling, the individual log_10_-transformed biomarker values for the four saliva samples (waking and bedtime samples on each of two days) were combined and used to compute participants’ diurnal cortisol intercepts (baseline cortisol at waking) and slopes (degree of change). Each participant’s cortisol intercept and slope were derived by fitting a linear regression line predicted from that person’s four cortisol values, accounting for variation in collection times and sampling days (see Adam and Gunnar, 2001; Hruschka et al., 2005; Adam, 2006; Saxbe et al., 2008). Given log_10_-transformed values, higher diurnal cortisol intercepts were indexed by less negative or more positive coefficients. Lower diurnal cortisol intercepts, indexed by more negative coefficients, were interpreted as representing a hypoactive HPA axis associated with decreased well-being (Rector et al., 2019) and greater stress (Vargas and Lopez-Duran, 2014). Diurnal cortisol slopes with more negative values (more steeply decreasing) were interpreted as representing more adaptive regulation of daily cortisol secretion (greater decreases in cortisol production from waking to bedtime), whereas flatter slopes with smaller negative values, or with zero or positive values, reflected less adaptive HPA axis functioning (Juster et al., 2010; Gunnar and Adam, 2012).

### Covariates

#### Outness

The Sexual Orientation Developmental Milestones questionnaire (Floyd and Stein, 2002) was used to assess participants’ degree of “outness” or sexual orientation disclosure. A single item *“Currently you are out to”* prompted participants to respond with options *1* = *No one* to *5* = *Most or all the significant people in your life.* This single item was used as a continuous variable with higher scores suggesting more outness.

### Analysis Plan

After examining zero-order correlations, we tested the total and direct associations of heterosexist and racist discrimination, and their interaction, with challenges to identity integration and salivary cortisol intercepts and slopes, as well as the indirect associations of discrimination with cortisol intercepts and slopes through challenges to identity integration (Hayes, 2015). We calculated six indirect effects by multiplying the effects of heterosexist discrimination and racist discrimination, and their interaction term on challenges to identity integration by the effect of challenges to identity integration on salivary cortisol intercepts and slopes. The indirect associations of heterosexist discrimination and racist discrimination, and their interaction term with salivary cortisol intercepts and slopes through challenges to identity integration were interpreted as meeting mediation criteria if the Bias-Corrected accelerated Confidence Intervals (BCa CIs) of the tested indirect effect, above and beyond the direct and total effects, did not include zero (Hayes, 2015) whether or not the direct and total effects were significant (Zhao et al., 2010; Rucker et al., 2011). The degree of model fit was assessed using the chi-square (χ^2^) goodness of fit statistic, comparative fit index (*CFI*, Bentler, 1990), Tucker-Lewis index (*TLI*, Tucker and Lewis, 1973), normed-fit index (*NFI*, Bentler and Bonnet, 1980), root mean square error of approximation (*RMSEA*; Browne and Cudeck, 1992), and standardized root mean square residual *(SRMR*). Model fit was acceptable if the χ^2^ statistic was non-significant (*p* > 0.05, Barrett, 2007), *CFI*, *TLI*, and *NFI* values were >0.95, and *RMSEA* and *SRMR* values were <0.06 and 0.08, respectively (Hu and Bentler, 1999).

The multiple mediation analysis was fitted as a path model in the lavaan package (Rosseel, 2012) in R, adjusting for all associated covariates in the model. We obtained 95% BCa CIs for the model’s estimates using bootstrapping (Preacher and Hayes, 2008; Mackinnon et al., 2010). We used bootstrapping because Shapiro–Wilk tests of normality showed that our main predictor variables, heterosexist and racist discrimination, were not normally distributed, all *p*s < 0.05, and because bootstrapping is robust against non-normality of the sampling distribution of the indirect effect (Montoya et al., 2017).

#### Missing Data

Missing data occurred at a low frequency, with 3.9% of data missing overall.^5^ Little’s MCAR test was not statistically significant, χ^2^(423) = 51.80, *p* > 0.05, thus, data were assumed to be at least missing at random. Considering the small amount of missing data and the small sample size, full information maximum likelihood was used to estimate missing data.

## Results

### Descriptive Analyses

Table 1 displays the means (*M*), standard deviations (*SD*), and zero-order correlations for all measures. Both heterosexist discrimination and racist discrimination were positively associated with challenges to identity integration. Challenges to identity integration was marginally negatively associated with salivary cortisol intercepts. Age was positively associated with outness and salivary cortisol intercepts. Outness was negatively associated with racist discrimination and challenges to identity integration.

**TABLE 1:** Descriptive statistics and zero-order correlations among main predictor, outcome, and control variables.

	***M***	***SD***	***Min***	***Max***	**1**	**2**	**3**	**4**	**5**	**6**	**7**
(1) Age	24.06	2.73	18.75	29.06	−	0.441**	–0.213	0.012	−0.237^†^	0.331*	–0.084
(2) Outness	4.16	1.17	2.00	5.00		−	–0.021	−0.276*	−0.436**	0.096	–0.032
(3) Heterosexist discrimination	1.20	1.25	0.00	4.00			−	0.199	0.346*	–0.037	–0.217
(4) Racist discrimination	2.16	0.73	1.12	4.47				−	0.351*	0.086	0.005
(5) Challenges to identity integration	3.93	1.45	1.00	6.83					−	−0.262^†^	–0.205
(6) Cortisol intercepts	0.010	0.113	–0.284	0.296						−	0.119
(7) Cortisol slopes	0.001	0.011	–0.027	0.025							−

N = 51; *p < 0.05; **p < 0.01; ^†^*p* < 0.10 (all tests were two tailed).

Participants who were assigned female at birth were younger (*M* = 23.27, *SD* = 2.85), *t*(49) = −2.28, *p* < 0.05, were less out (*M* = 3.78, *SD* = 1.31), *t*(49) = −2.58, *p* < 0.05, reported more heterosexist discrimination (*M* = 1.56, *SD* = 1.28), *t*(49) = 2.27, *p* < 0.05, and reported more challenges to identity integration (*M* = 4.37, *SD* = 1.51), *t*(48) = 2.32, *p* < 0.05 when compared to participants who were assigned male at birth (*Ms* = 24.95, 4.58, 0.79, 3.45; *SDs* = 2.34, 0.83, 1.10, 1.62, respectively). Analyses of variance with Bonferroni-corrected *post hoc* tests showed that self-identified bisexual/pansexual participants were marginally younger, *F*(3,47) = 2.98, *p* < 0.05 (*M* = 23.05, *SD* = 3.12), less out, *F*(3,47) = 7.25, *p* < 0.001 (*M* = 3.29, *SD* = 1.26), and reported greater challenges to identity integration, *F*(3,46) = 3.16, *p* < 0.05 (*M* = 4.68, *SD* = 1.25) than self-identified gay participants (*Ms* = 25.35, 4.83, 3.23; *SDs* = 2.03, 0.51, 1.62). There were no other significant differences among the sexual orientation groups. Participants’ age, sex assigned at birth, ethnicity/race (monoethnic/racial or biracial), and outness were included as covariates in the subsequent analyses. The zero-order correlations between heterosexist discrimination and racist discrimination, and between salivary cortisol intercepts and slopes, were both non-significant, and the study did not have a goal of examining such associations; therefore, these associations were not covaried in the model.

### Associations Between Heterosexist and Racist Discrimination, Challenges to Identity Integration, and Salivary Diurnal Cortisol Intercepts and Slopes

**FIGURE 1:**
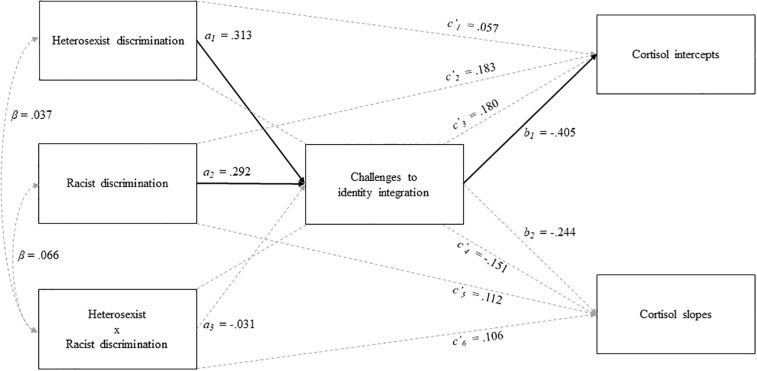
Mediation path analysis. Solid lines represent statistically significant paths. Dashed lines represent non-significant paths. β represents standardized coefficients. Unstandardized coefficients are provided in Table 2.

**FIGURE 2:**
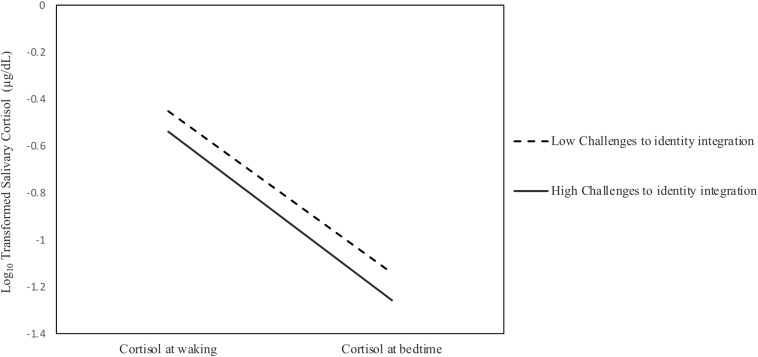
Averaged waking and bedtime salivary cortisol at low and high (±1 SD) Challenges to identity integration.

The unstandardized regression coefficients for total, direct, and indirect effects for the full model are reported in Table 2, and to aid interpretation, standardized coefficients are presented in Figure 1. Overall, the model showed good fit to the data (χ^2^ = 2.07, *df* = 2, *p* > 0.05; χ^2^/*df* = 1.04; *CFI* = 0.999; *TLI* = 0.969; *NFI* = 0.978 *RMSEA* = 0.026, 90% BCa CIs = [0.000,0.280], *SRMR* = 0.032), accounting for 18.1, 11.3, and 18.3% of the variability in salivary cortisol intercepts, diurnal slopes, and challenges to identity integration, respectively. Contrary to the first hypothesis, the total and direct effects of heterosexist discrimination (*c*_1_), racist discrimination (*c*_2_) and the interaction term between heterosexist and racist discrimination (*c*_3_) on salivary cortisol intercepts and diurnal cortisol slopes were non-significant. Supporting the second hypothesis, heterosexist discrimination (*a*_1_) and racist discrimination (*a*_2_) were both positively associated with challenges to identity integration. Partially supporting the third hypothesis, challenges to identity integration was significantly negatively associated with salivary cortisol intercepts (*b*_1_) but was not associated with diurnal cortisol slopes (*b*_2_) (see Figure 2).

**TABLE 2:** Multiple mediation analysis.

**Outcome**	**Predictor**	**Label**	***EST***	***SE***	***LCI***	***UCI***
Cortisol intercepts						
	Heterosexist discrimination	*c’_1_*	0.005	0.013	–0.023	0.028
	Racist discrimination	*c’_2_*	0.028	0.024	–0.018	0.080
	Heterosexist by racist discrimination	*c’_3_*	0.023	0.018	–0.009	0.063
	Challenges to identity integration	*b*_1_	–0.063	0.025	**−0.116**	**−0.018**
Cortisol slopes						
	Heterosexist discrimination	*c’_4_*	–0.001	0.002	–0.004	0.002
	Racist discrimination	*c’_5_*	0.002	0.002	–0.002	0.006
	Heterosexist by racist discrimination	*c’_6_*	0.001	0.002	–0.002	0.005
	Challenges to identity integration	*b*_2_	–0.004	0.002	–0.008	0.001
Challenges to identity integration						
	Heterosexist discrimination	*a*_1_	0.184	0.075	**0.039**	**0.333**
	Racist discrimination	*a*_2_	0.295	0.140	**0.010**	**0.532**
	Heterosexist by racist discrimination	*a*_3_	–0.026	0.116	–0.286	0.197
**Covariances**						
Heterosexist discrimination						
	Heterosexist by racist discrimination		0.040	0.166	–0.240	0.428
Racist discrimination						
	Heterosexist by racist discrimination		0.042	0.198	–0.259	0.534
**Indirect effects for cortisol intercepts**						
	Heterosexist discrimination → challenges to identity integration → cortisol intercepts	*a*_1_*b_1_	–0.011	0.008	**−0.032**	**−0.001**
	Racist discrimination → challenges to identity integration → cortisol intercepts	*a*_2_*b_1_	–0.018	0.013	**−0.055**	**−0.002**
	Heterosexist by racist discrimination → challenges to identity integration → cortisol intercepts	*a*_3_*b_1_	0.002	0.008	–0.012	0.023
**Total effects for cortisol intercepts**						
	*c’_1_* + (*a*_1_*b_1_)	*c*_1_	–0.006	0.013	–0.035	0.018
	*c’_2_* + (*a*_2_*b_1_)	*c*_2_	0.010	0.021	–0.031	0.055
	*c’_3_* + (*a*_3_*b_1_)	*c*_3_	0.025	0.017	–0.008	0.064
**Indirect effects for cortisol slopes**						
	Heterosexist discrimination → challenges to identity integration → cortisol slopes	*a*_1_*b_2_	–0.001	0.001	–0.002	1.35E–04
	Racist discrimination → challenges to identity integration → cortisol slopes	*a*_2_*b_2_	–0.001	0.001	–0.004	9.48E–05
	Heterosexist by racist discrimination → challenges to identity integration → cortisol slopes	*a*_3_*b_2_	9.17E–05	0.001	–0.001	0.002
**Total effects for cortisol slopes**						
	*c’_4_* + (*a*_1_*b_1_)	*c*_4_	–0.002	0.002	–0.005	0.001
	*c’_5_* + (*a*_2_*b_1_)	*c*_5_	0.001	0.002	–0.003	0.004
	*c’_6_* + (*a*_3_*b_1_)	*c*_6_	0.001	0.002	–0.002	0.005

*LCI and UCI: lower confidence interval and upper confidence interval (bias-corrected accelerated 95%); 10,000 bootstrap samples. Significant BCa CIs are presented in bold font.*

Tests of indirect effects were used to evaluate the fourth hypothesis. As predicted, both the specific indirect effect of heterosexist discrimination on salivary cortisol intercepts through challenges to identity integration (*a*_1_^∗^*b*_1_) and the specific indirect effect of racist discrimination on salivary cortisol intercepts through challenges to identity integration (*a*_2_^∗^*b*_1_) were significant. The corresponding indirect effects for diurnal cortisol slopes were non-significant, nor were there significant indirect effects of the interaction term between heterosexist and racist discrimination on salivary cortisol intercepts or diurnal cortisol slopes through challenges to identity integration. Thus, sexually diverse Latinx emerging adults’ experiences of greater heterosexist and racist discrimination were associated with lower salivary cortisol intercepts through their having more challenges to identity integration.

## Discussion

Studies of neurobiological functioning have yet to consider how two or more forms of oppression, such as heterosexist and racist discrimination, interconnect to marginalize social groups like sexually diverse Latinx emerging adults, undermining their physiological and psychosocial adjustment and conferring health risks. At the same time, few studies of intersectionality have considered the neurobiological impacts of experiencing complexly intertwined discrimination targeted toward multiple social categorical identities within social groups at various social locations. We sought to bridge these gaps through the application of an intersectional lens (Crenshaw, 1989) to models of minority stress (e.g., Meyer, 2003; Myers, 2009) and allostatic load (McEwen and Stellar, 1993) to further understand the lived experiences of sexually diverse Latinx people during emerging adulthood, which is a developmental period associated with sexual and ethnic/racial identity exploration, formation, and consolidation processes implicated with people’s ability to healthfully adjust (Arnett, 2004). Experiences of heterosexist and racist discrimination in sexually diverse Latinx people during emerging adulthood are associated with a host of social inequalities and inequities that perpetuate health disparities (Rhodes et al., 2020). Moreover, heterosexist and racist social contexts can challenge sexually diverse Latinx emerging adults from integrating their multiple marginalized social group identities, and these challenges are associated with psychological distress and depressive symptoms (Morales, 1989; Espín, 1993; Santos and VanDaalen, 2016; Shramko et al., 2018).

In the current study, we specifically examined the additive and multiplicative effects of heterosexist and racist discrimination on sexually diverse Latinx emerging adults’ diurnal adrenocortical functioning and challenges with integrating their multiple marginalized social identities. Heterosexist discrimination and racist discrimination were each positively associated with challenges to identity integration, suggesting that single-axis measures of discrimination independently and additively associate with challenges to identity integration among sexual diverse Latinx emerging adults. Healthy sexual and ethnic/racial identity integration is a developmental process crucial for the well-being of sexually and ethnically/racially diverse emerging adults (Shramko et al., 2018). Heterosexist discrimination is associated with hesitation to identify as a sexually diverse group member (Herek and Garnets, 2007) and with developing negative attitudes and beliefs of the self (i.e., internalized homonegativity) (Igartua et al., 2003; Herek et al., 2009). Racist discrimination has been associated with confusion about and lack of sense of belonging with a Latinx ethnic/racial group membership (Umaña-Taylor et al., 2008). In parallel with our findings, Shramko et al. (2018) also reported that sexually diverse Latinx emerging adults experience more challenges to the integration of their sexual and ethnic/racial social group identities when they report more heterosexist and racist discrimination (Shramko et al., 2018).

For sexually diverse Latinx emerging adults, cultural factors such as the importance of family, traditional gender roles, conservative religious values, and widespread heterosexism within Latinx culture are described as potential stressors associated with challenges to the healthy integration of their sexually diverse and Latinx social group identities (Espín, 1993; Rodriguez, 1996; Díaz, 2013). Moreover, experiences of racist discrimination within mainstream sexually diverse White communities have also been associated with challenges to sexual and ethnic/racial identity integration in sexually diverse Latinx people (Savin-Williams, 1996). Thus, it is likely that discriminatory heterosexist and racist contexts challenge sexually diverse Latinx emerging adults’ capacity to navigate and integrate their multiple marginalized social group identities, and these challenges have been associated with diminished well-being and poor health in sexually diverse people of color (Sarno et al., 2015; Santos and VanDaalen, 2016; Shramko et al., 2018).

Yet, heterosexist and racist discrimination were not directly associated with adrenocortical diurnal functioning, neither additively nor multiplicatively (their interaction term). The statistically non-significant total and direct effects for heterosexist and racist discrimination on adrenocortical patterns in this study suggest that single-axis (i.e., assessing multiple marginalized social identities and/or experiences independently), additive (i.e., the sum of multiple marginalized social identities and/or experiences), and multiplicative (interactions among multiple marginalized social identities and/or experiences) effects may not always capture the complex processes by which two or more systems of oppression, specifically heterosexist and racist discrimination, intersect (Bowleg, 2008) and “get under the skin” to confer elevated risk for health disparities. Rather, it was the individuals’ internalized, subjective and personally relevant processing of these multi-faceted discriminatory and oppressive experiences that accounted for their compromised adrenocortical activity (Parra and Hastings, 2018).

More specifically, we found significant indirect effects suggesting that challenges to identity integration is a plausible intersectional mechanism by which heterosexist and racist discrimination create social disparities in physical health. Sexually diverse Latinx emerging adults’ feelings of being more challenged with identity integration significantly linked the associations of both more heterosexist discrimination and more racist discrimination with hypoactive adrenocortical activity, as indicated by reduced salivary cortisol intercepts. Thus, challenges to identity integration may be a form of internalized social distress that compromises adaptive adrenocortical regulation. Lower cortisol intercepts, indicative of less circulating cortisol at waking, have been associated with diminished psychological health (Rector et al., 2019) and are considered an index of dysregulated adrenocortical functioning associated with exposure to severe and pervasive stress (Adam and Kumari, 2009; Vargas and Lopez-Duran, 2014). This interpretation suggests that experiences at the intersections of heterosexist and racist discrimination may influence sexually diverse Latinx emerging adults to keep their sexual and ethnic/racial marginalized social group identities separate from each other, and that this experience of one’s self living between divided social worlds (Morales, 1989) carries a physical, biological toll.

It is important to note that challenges to identity integration should not be considered a proxy for the intersection of racist and heterosexist discrimination. Rather, challenges to identity integration reflects a psychological, intrapersonal identity process that may be exacerbated by experiences of discrimination related to sexually diverse Latinx people’s multiply marginalized social locations. This perspective reinforces the necessity of examining within-group heterogeneity of social group memberships, which is critical for the application of an intersectionality lens in quantitative research (McCall, 2005; Cole, 2009; DeBlaere et al., 2010; Parent et al., 2013; Else-Quest and Hyde, 2016a, b). Doing so requires the development and use of inherently intersectional measures (Cole, 2009; DeBlaere et al., 2010; Balsam et al., 2011; Sarno et al., 2015; Else-Quest and Hyde, 2016b) of multifaceted identity processes in order to gain deeper insights into the links between complex experiences of specific and systemic oppression within systems of power and privilege and individual manifestations of compromised health and well-being in quantitative studies.

Unexpectedly, heterosexist discrimination, racist discrimination, and challenges to identity integration were not associated with diurnal salivary cortisol slopes. Previous studies have documented associations between experiencing more heterosexist discrimination and manifesting flatter diurnal slopes in sexually diverse White Canadian emerging adults (Parra et al., 2016) and shown that Black U.S. gay and bisexual men have flattened and less variable diurnal cortisol output than White U.S. gay and bisexual men (Cook et al., 2017). In addition to the differences in sample demographics, these prior studies also differed from the current study in both the number of samples used to measure diurnal slopes and the analytical approach used to calculate diurnal slopes, which can affect findings (Stewart and Seeman, 2000; Stalder et al., 2016). Whether these methodological differences may account for the current study’s lack of direct and total significant associations with diurnal cortisol slopes, or whether this may be specific to the links between adrenocortical functioning, identity integration, and discrimination experiences for sexually diverse Latinx emerging adults is unclear. It would be advantageous for future studies to collect more salivary samples over multiple points of two or more days while collecting more objective measures of waking and bedtime sampling times (Granger et al., 2012), including quality of sleep (Backhaus et al., 2004), in order to have greater opportunity to capture the dynamic fluctuations and degree of change of daily salivary cortisol output.

The interpretation of these findings should be taken with caution. Causality and directionality of associations cannot be proven because this study used a non-experimental and cross-sectional design. Additionally, the study included a convenience sample of participants from a particular social categorical community such that findings should not be assumed to be generalizable to other multiply marginalized social categorical groups. Although comparably sized samples have been sufficient for detecting direct associations between discrimination and biological measures in prior studies (e.g., Parra et al., 2016), the sample size may have precluded our ability to detect total and direct associations of discrimination on cortisol slopes and intercepts. It is important to note that although the current study may have been underpowered to detect direct and total effects, the reported indirect effects were robust after accounting for participants’ age, sex assigned at birth, monoethnic/racial and biracial identities, and outness.

Additionally, prospective studies would substantially help to advance research in this area. Longitudinal designs may potentially reveal the directionality or temporal order of effects between experiences of heterosexist and racist discrimination on adrenocortical functioning through challenges to identity integration, particularly during emerging adulthood as it is a developmental period associated with identity confusion, exploration, and integration (Arnett, 2000, 2001, 2006). Indeed, prospective research has shown enduring effects of racist discrimination on adrenocortical functioning (Adam et al., 2015) and of heterosexist and racist discrimination on developing positive sexual and ethnic/racial identities, respectively (Pahl and Way, 2006; Patterson, 2008; Umaña-Taylor et al., 2008). Whether there are lasting effects of heterosexist and racist discrimination on identity integration and adrenocortical functioning in sexually diverse Latinx emerging adults, or other sexually diverse people of color, remains to be seen.

## Conclusion

Sexually diverse Latinx emerging adults are tasked with consolidating a clear sense of self that integrates their multiple intersecting social group memberships (Espín, 1993; Morales, 1989) during an important development period of both opportunity and vulnerability (Arnett, 2007; Morgan, 2013). Yet, sexually diverse Latinx people experience considerable amounts of discrimination based on their marginalized social group memberships (Rhodes et al., 2020) that negatively impacts their healthy neurobiological and psychosocial adjustment (Parra and Hastings, 2018). The complex associations between systems and processes of oppression and aspects of sexuality (attraction, behavior, and identity) and ethnicity/race (language, culture, and identity) are not sufficiently well understood to help explain health and psychosocial disparities in sexually diverse people of color. Drawing from the available adrenocortical and identity integration literature in both sexually diverse and Latinx groups of people, it is likely that the lived experiences of discrimination faced by sexually diverse Latinx emerging adults, which are situated within multiple systems of power and privilege, adversely impacts their adrenocortical stress responses and their ability to integrate their marginalized social identities. Challenges to identity integration may represent one way in which heterosexism and racism intersect to socially and biologically disadvantage sexually diverse people of color by tasking them to integrate their divided social worlds.

Our work showed that measures that are inherently intersectional (i.e., assessing how sexually diverse people of color integrate their sexual and ethnic/racial identities in the context of heterosexism and racism) are necessary to identify processes unique to the intersections of multiple forms of oppression that constitute health disparities in sexually diverse Latinx emerging adults. Experiences of heterosexist and racist discrimination were indirectly associated with atypical daily cortisol patterns through challenges with identity integration through additive, but not multiplicative, processes. Conceptualizing the intersection of two forms of oppression via statistical interactions did not directly capture how sexually diverse Latinx people are physiologically and psychologically impacted by heterosexism and racism, whereas an inherently intersectional measure, the challenges in allegiances scale (Sarno et al., 2015), did capture these effects (Parra and Hastings, 2018). Thus, heterosexist and racist discrimination may intertwine to disenfranchise sexually diverse Latinx emerging adults from thriving both within and across their marginalized social groups, as evidenced through dysregulated patterns in salivary cortisol production and challenges to identity integration, both of which are essential socio-biological processes for healthy physical and social adaptation.

## Data Availability Statement

The raw data supporting the conclusions of this article will be made available by the authors to any qualified researcher upon request.

## Ethics Statement

This study was carried out in accordance with the recommendations of and approved by the UC Davis Institutional Review Board. All participants gave written informed consent.

## Author Contributions

LP conceptualized the study design and analyses, executed data collection, cleaning, scoring, and statistical analyses, interpreted the results, drafted and revised the manuscript, provided final approval of the version to be published, and takes accountability for all aspects of the work, including accuracy and validity of the contents. PH conceptualized the study design and analyses, provided interpretation of the results, drafted and revised the manuscript, provided final approval of the version to be published, and takes accountability for all aspects of the work, including accuracy and validity of the contents.

## Conflict of Interest

The authors declare that the research was conducted in the absence of any commercial or financial relationships that could be construed as a potential conflict of interest.

## Footnotes

1 Although biracial Latinx-White people are known to experience unique forms of discrimination and challenges to their ethnic and/or racial identity integration (Jackson et al., 2012; Soliz et al., 2017), *t*-tests showed no mean differences between monoethnic Latinx and biracial Latinx-White people among measures of sexual and ethnic discrimination, identity integration, and salivary cortisol indices (all *p*s > 0.05). Given the small number of biracial Latinx-White people in this study, in an effort to yield more precise estimates, participants’ monoethnic and biracial identities were included as a covariate in the main analyses.

2 The frequent use of glucocorticoids for asthmatic patients has been shown to decrease morning cortisol (Masharani et al., 2005), yet more recent evidence suggests that glucocorticoid dosage does not affect cortisol concentrations for asthmatic patients who adhere to their prescribed glucocorticoid doses (Shin et al., 2014). In the present study, participants were asked to refrain from using their glucocorticol steroids two days before and during the two days of data collection. The use of all medications, including hormones, was tested for their associations with raw waking and evening salivary cortisol samples prior to computing salivary cortisol intercepts and slopes, all *p*s > 0.05; please see section “Measures.”

3 The term “racist discrimination” encompasses discrimination based on a person’s cultural heritage, phenotype, language, and/or membership to an ethnic group, all which fall within the broader definition of racism (Jones, 2001).

4 Independent sample *t*-tests showed no mean differences in salivary cortisol between participants who endorsed the use of medication (*M* = 0.24, *SD* = 0.23), *t*(196) = −0.197, *p* > 0.05; experienced unusual events (*M* = 0.25, *SD* = 0.22), *t*(196) = −0.401, *p* > 0.05, and who reported consuming food (*M* = 0.18, *SD* = 0.16), *t*(195) = 0.78, *p* > 0.05, or tobacco (*M* = 0.20, *SD* = 0.02), *t*(196) = 0.257, *p* > 0.05, when compared to participants who did not (*M* = 0.23, 0.23, 0.23, 0.23; *SD* = 0.22, 0.21, 0.22, 0.22, respectively). All participants reported that they did not consume caffeine 1 h prior to sampling.

5 Participants returned six frozen saliva samples, which included two waking and two bedtime samples that were used to compute cortisol intercepts and slopes for this study. Of the 204 total waking and bedtimes samples (four samples per participant), 2.9% (*n* = 6) of oral swabs did not have sufficient saliva for assay; and four of these six oral swabs with insufficient saliva belonged to a single participant. The participant was retained in the study and their missing cortisol intercepts and slopes were estimated using full information maximum likelihood.
